# Akkermansia muciniphila inhibited the periodontitis caused by Fusobacterium nucleatum

**DOI:** 10.1038/s41522-023-00417-0

**Published:** 2023-07-17

**Authors:** Bingqing Song, Wenpan Xian, Yan Sun, Lichen Gou, Qiang Guo, Xuedong Zhou, Biao Ren, Lei Cheng

**Affiliations:** 1 State Key Laboratory of Oral Diseases, West China Hospital of Stomatology, National Clinical Research Center for Oral Diseases, Sichuan University, 610041 Chengdu, China; 2 Department of Operative Dentistry and Endodontics, West China School of Stomatology, Sichuan University, 610041 Chengdu, China

**Keywords:** Pathogens, Plaque

## Abstract

Periodontitis is the most important cause of tooth loss in adults and is closely related to various systemic diseases. Its etiologic factor is plaque biofilm, and the primary treatment modality is plaque control. Studies have confirmed that *Fusobacterium nucleatum* can cause periodontitis through its virulence factors and copolymerizing effects with other periodontal pathogens, such as the red complex. Inhibiting *F. nucleatum* is an essential target for preventing periodontitis. The time-consuming and costly traditional periodontal treatment, periodontal scaling, and root planing are a significant burden on individual and public health. Antibiotic use may lead to oral microbial resistance and microbiome imbalance, while probiotics regulate microbial balance. *Akkermansia muciniphila* is a critical probiotic isolated from the human intestine. It can protect the integrity of the epithelial barrier, regulate and maintain flora homeostasis, improve metabolism, and colonize the oral cavity. Its abundance is inversely correlated with various diseases. We hypothesized that *A. muciniphila* could inhibit the effects of *F. nucleatum* and alleviate periodontitis. Bacterial co-culture experiments showed that *A. muciniphila* could inhibit the expression of the virulence gene of *F. nucleatum*. After treating gingival epithelial cells (GECs) with *F. nucleatum* and *A. muciniphila*, transcriptome sequencing and ELISA experiments on medium supernatant showed that *A. muciniphila* inhibited the inflammatory effect of *F. nucleatum* on GECs by inhibiting TLR/MyD88/NF-κB pathway modulation and secretion of inflammatory factors. Finally, animal experiments demonstrated that *A. muciniphila* could inhibit *F. nucleatum*-induced periodontitis in BALB/c mice.

## Introduction

Periodontitis occurs in periodontal supporting tissues and is a chronic inflammatory disease that most commonly causes tooth loss in adults^1^. The Global Burden of Disease study shows severe periodontitis has become the sixth most prevalent disease worldwide^2^. At the same time, periodontitis is associated with various systemic diseases.

Dental plaque is the initiating factor of periodontitis^3^. Disrupting the balance between dental plaque and the host immunity leads to periodontal inflammation.

*Fusobacterium nucleatum* is a gram-negative obligate anaerobic bacterium and is a typical representative of the orange complex in periodontal pathogens. It is highly detected in periodontal diseases, and its detection positively correlates with periodontal inflammation^4,5^. *F. nucleatum* alone infecting mice can lead to significant periodontal inflammation and alveolar bone destruction^6^. It can promote the formation and maturation of plaque biofilm through a copolymerizing effect and the occurrence and development of periodontitis in conjunction with the red complex through virulence factors FadA, endotoxins, etc^7–9^. In addition, it mediates the relationship between periodontitis, colorectal cancer, and other systemic diseases^10–12^. Inhibiting its growth and virulence gene expression is significant for periodontal and general health.

Traditional periodontal treatment is based on periodontal scaling and root planing^13^, supplemented by antibiotics if necessary^14,15^. It is costly, time-consuming, and a massive burden on individual and public health because it may not eradicate the plaque and lead to microbial resistance and microbiome imbalance^16–18^. Therefore, researchers have been trying to find new methods to control plaque in recent years.

Probiotics can produce antibacterial products, regulate the host’s immune response, and interact with pathogenic bacteria directly. They have been introduced into the study of periodontitis treatment to inhibit the progression of periodontitis in recent years^19^. *Akkermansia muciniphila*, also known as Akk, is a gram-negative obligate anaerobic bacterium that accounts for about 3% of the microbial community in the colon of healthy people^20,21^. Its abundance is inversely correlated with various diseases^22–29^ and is an intestinal probiotic that has attracted widespread attention in recent years^30,31^. It can protect the integrity of the epithelial barrier, regulate and maintain intestinal flora homeostasis, and improve metabolism^32–34^.

Previous studies have shown that *A. muciniphila* sequence could be detected in saliva^35^, and oral administration of 10^10^ live or pasteurized *A. muciniphila* bacteria daily is safe^24^. *A. muciniphila* can colonize the oral cavity and may be used as a probiotic for oral niches.

Our study found that *A. muciniphila* can inhibit the growth and virulence gene expression of *F. nucleatum* and reduce TLR/MyD88/NF-κB pathway expression and inflammatory factors upregulated by *F. nucleatum* to suppress GECs’ inflammatory response. It can also reduce alveolar bone loss and soft tissue inflammatory response in *F. nucleatum*-induced mice periodontitis.

## Results

### *A. muciniphila* inhibits the growth of *F. nucleatum* in a planktonic or biofilm state

Researchers commonly use the BHI-Hemin-VK medium to culture *F. nucleatum* and the BHI-Mucin medium for *A. muciniphila*. We found that both usually grow in the BHI-Hemin-VK medium (Supplementary Fig. 1a, b), while *F. nucleatum* proliferates at a low rate and rapidly dies in the BHI-Mucin medium (Supplementary Fig. 1b). We finally chose the BHI-Hemin-VK medium as the co-culture medium.

**Fig. 1:**
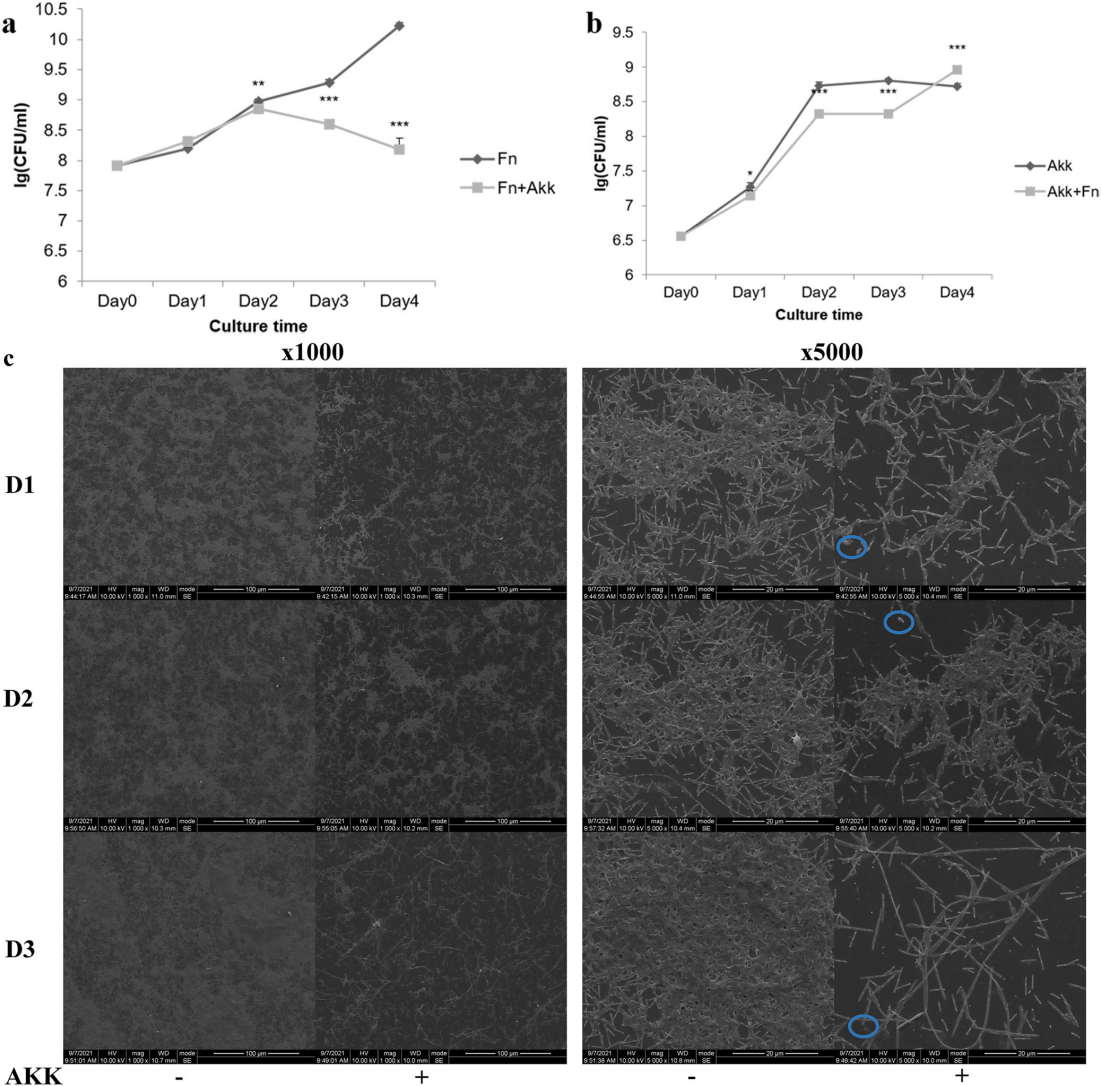
*A. muciniphila* inhibited the growth of *F. nucleatum* in the planktonic or biofilm state. **a** The amount of *F. nucleatum* when *F. nucleatum* and *A. muciniphila* were co-cultured. **b** The amount of *A. muciniphila* when *F. nucleatum* and *A. muciniphila* were co-cultured. **c** Formation of biofilms when *F. nucleatum* was cultured alone and co-cultured with *A. muciniphila* using electron microscopic scanning (SEM) (×1000 and ×5000), scale = 100 μm and 200 μm, AKK −: *F. nucleatum* cultured alone; AKK + : *F. nucleatum* co-cultured with *A. muciniphila*; D1, D2, and D3: biofilms formed by each group on days 1, 2, and 3; blue circle: *A. muciniphila* under SEM. Data are shown as the mean ± SD. Statistical significance was determined by Student–Newman–Keuls test. *P < 0.05, **P < 0.01, ***P < 0.001, ns not significant.

To explore how *A. muciniphila* influences the growth of *F. nucleatum*, we co-cultured them to observe the amounts of bacteria in the planktonic and biofilm states. In the planktonic state, *F. nucleatum* could not grow to the plateau stage, and its amount was significantly lower than the Fn group in 2–4 days; its growth was significantly inhibited, leading to gradual death (Fig. 1a). *A. muciniphila* could grow to the plateau stage (approximately 1 × 10^8^ CFU/mL) in both the AKK and Fn+AKK groups and the bacterial proliferation was closer on days 3–4. However, the final bacterium amount was different (Fig. 1b). Scanning electron microscopy (S-ES) showed that the amount of *F. nucleatum* biofilm increased significantly over time in the Fn group, while the amount of biofilm in the co-culture group did not change significantly on day 1 but slightly increased on day 2. Furthermore*, A. muciniphila* significantly inhibited *F. nucleatum* biofilm formation and growth on days 1–3, consistent with the results of the planktonic state. *F. nucleatum* was elongated and filamentous, with poor growth status on day 3 (Fig. 1c).

### *A. muciniphila* inhibited the expression of *F. nucleatum* virulence genes

**Fig. 2:**
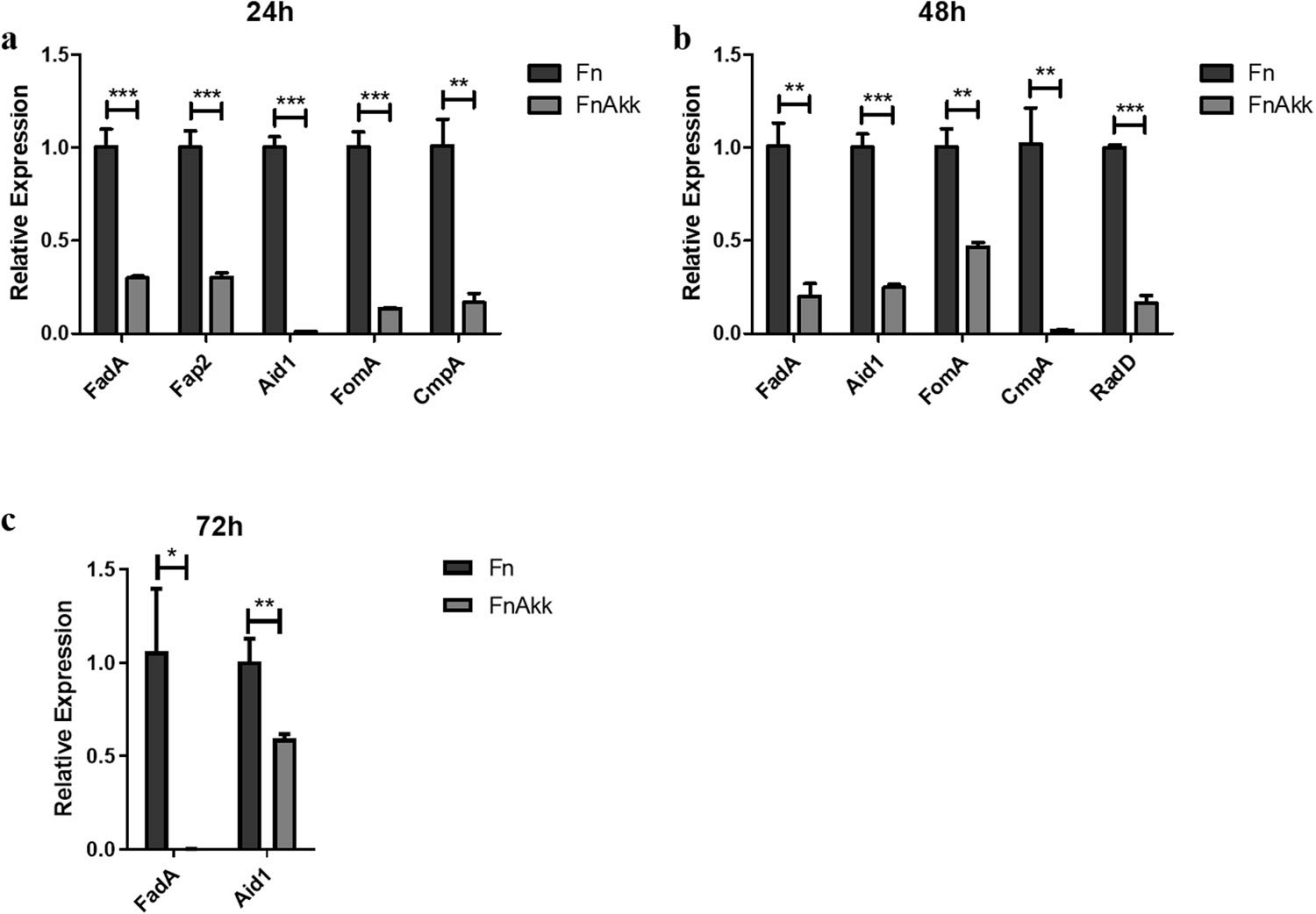
*A. muciniphila* inhibited the expression of *F. nucleatum* virulence genes. **a** FadA, Fap2, Aid1, FomA, and CmpA gene expression of *F. nucleatum* in 24 h. **b** FadA, Aid1, FomA, CmpA, and RadD gene expression of *F. nucleatum* in 48 h. **c** FadA and Aid1 gene expression of *F. nucleatum* in 72 h. Data are shown as the mean ± SD. Statistical significance was determined by Student–Newman–Keuls test. *P < 0.05, **P < 0.01, ***P < 0.001, ns not significant.

The pathogenicity of *F. nucleatum* is associated with various virulence factors. It releases virulence factors such as FadA, endotoxins, and serine proteases^36^ to induce host immune-inflammatory responses and promotes periodontal tissue destruction^37^. We further explored whether *A. muciniphila* influenced *F. nucleatum* virulence gene expression. We extracted the RNA in the Fn and Fn+Akk groups the days 1, 2, and 3 to quantify gene expression of *F. nucleatum* with qPCR. We found that FadA, Fap2, Aid1, FomA, and CmpA genes were significantly inhibited on day 1 (Fig. 2a), FadA, Aid1, FomA, CmpA, radD genes were markedly downregulated on day 2 (Fig. 2b), FadA and Aid1 genes were still significantly lower on day 3 than in the Fn group (Fig. 2c). It follows that *A. muciniphila* inhibited the virulence gene expression of *F. nucleatum*.

### *A. muciniphila* inhibited the inflammatory effect on gingival epithelial cells caused by *F. nucleatum* by inhibiting the expression of TLR/MyD88/NF-κB pathways and secretion of inflammatory factors

*A. muciniphila* can inhibit *F. nucleatum* growth and its virulence gene expression. However, to determine whether *A. muciniphila* could block the inflammatory effect on GECs caused by *F. nucleatum* to inhibit periodontitis, we infected human GECs (C1052) with *A. muciniphila* and *F. nucleatum* alone and together (control, Fn, AKK, and Fn+Akk groups, multiplicity of infection (MOI) = 250) to further clarify the role of *A. muciniphila*.

RNA-seq of GECs showed that in the Fn group, 1407 and 563 genes were downregulated, and 1429 and 756 genes were upregulated, respectively, compared with the control and the Fn+AKK groups (Supplementary Fig. 2a). *F. nucleatum* and *A. muciniphila* affected the gene expression of GECs differently.

Kyoto Encyclopedia of Genes and Genomes (KEGG) functional enrichment analysis showed significant changes in cytokine-cytokine receptor interaction, JAK-STAT signaling pathway, TNF signaling pathway, etc., in the Fn group compared with the control group (Supplementary Fig. 2b). In addition, the TNF signaling pathway, cytokine-cytokine receptor interaction, etc., changed in the Fn+Akk group compared with the Fn group (Supplementary Fig. 2b). These findings illustrated that *F. nucleatum* alone and *F. nucleatum* and *A. muciniphila* co-infection affected the expression of inflammatory factors in human GECs differently.

Go enrichment yielded similar results: there were significant changes in the cell surface receptor signaling pathway, etc., compared with the control group, and the cytokine-mediated signaling pathway, cell surface receptor signaling pathway, etc., exhibited significant changes in the Fn+Akk group compared with the Fn group (Supplementary Fig. 2c).

**Fig. 3:**
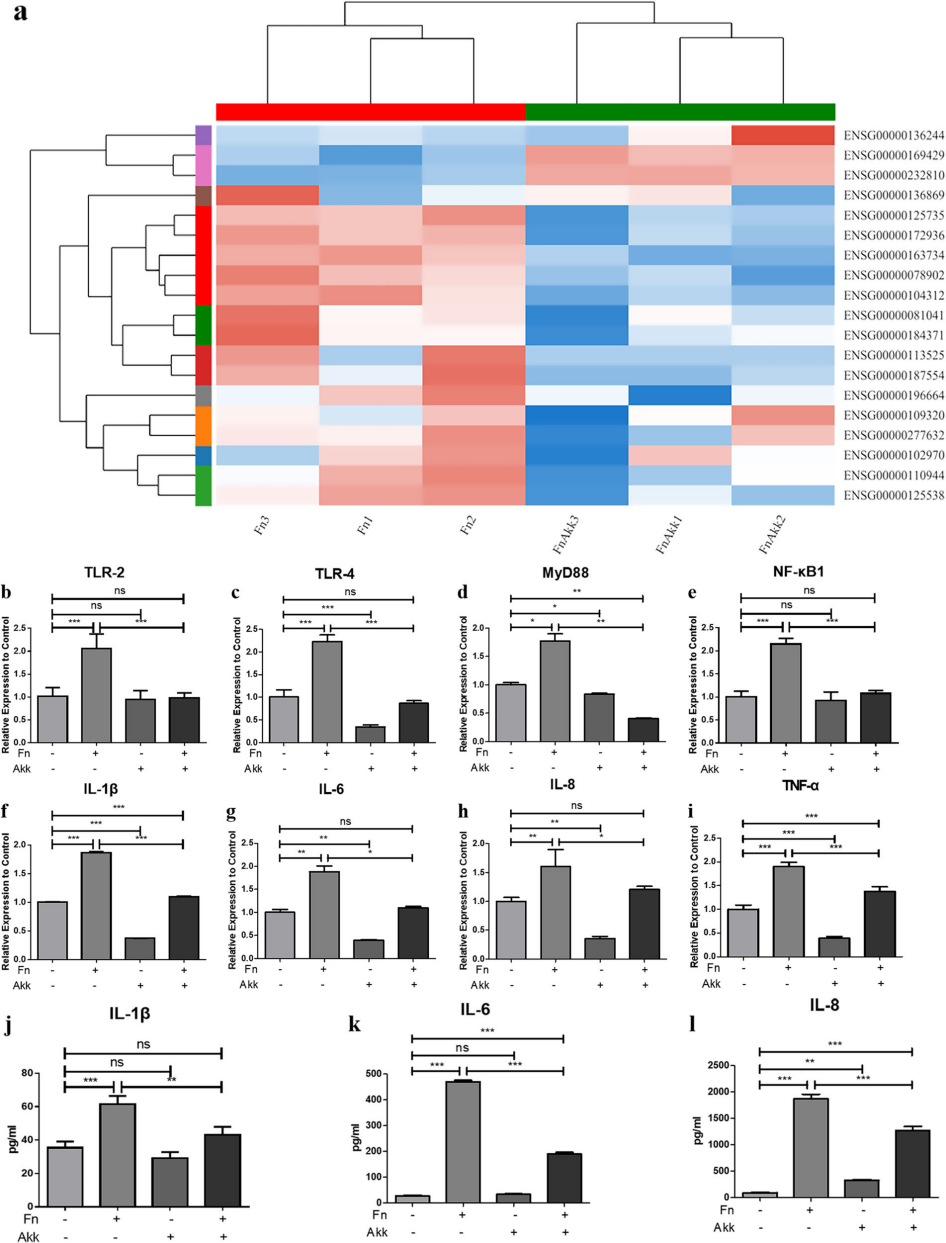
*A. muciniphila* inhibited the inflammatory effect on gingival epithelial cells caused by *F. nucleatum* by inhibiting the expression of TLR/MyD88/NF-κB pathways and secretion of inflammatory factors. **a** Cluster analysis of inflammatory factor genes of human GECs treated by bacteria for 24 h (ordinate: gene number and name, abscissa: sample name, color indicates the expression of genes in this sample, red represents higher expression, blue represents lower expression). **b**–**i** Expression of TLR-2 (**b**), TLR-4 (**c**), MyD88 (**d**), NF-κB1 (**e**), IL-1β (**f**), IL-6 (**g**), IL-8 (**h**), and TNF-α (**i**) of human GECs infected for 24 h; **j**–**l** Secretion of IL-1β (**j**), IL-6 (**k**), and IL-8 (**l**) by human GECs infected for 24 h. Data are shown as the mean ± SD. Statistical significance was determined by One-way analysis of variance (ANOVA) and Student–Newman–Keuls test. *P < 0.05, **P < 0.01, ***P < 0.001, ns not significant.

Cluster analysis of inflammatory factors-related genes showed that inflammatory factors such as TLRs (ENSG00000136869, ENSG00000187554, ENSG00000109320)/MyD88(ENSG00000172936)/NF-κB (ENSG00000109320) pathway and IL-1β (ENSG00000125538) in the Fn+AKK group were downregulated significantly reduced compared with the Fn group (Fig. 3a). qPCR of representative factors of the NF-κB pathway and inflammatory factors of periodontitis^38^ showed that TLR-4, MyD88, IL-1β, IL-6, IL-8, and TNF-α were significantly downregulated in the AKK group, but not TLR-2 and NF-κB1, compared with the control group (Fig. 3b–i). In the Fn group, MyD88 and four inflammatory factors increased significantly; TLR-2, TLR-4, and NF-κB1 were expressed twice or more as high as those in the control group (Fig. 3b–i). In the Fn+Akk group, TLR-2, TLR-4, MyD88, and NF-κB1 were expressed approximately half as much as those in the Fn group, and inflammatory factors were expressed significantly lower than those in the Fn group (Fig. 3b–i). These indicated that *A. muciniphila* downregulated the TLR/MyD88/NF-κB pathway and inflammatory factors expression of human GECs caused by *F. nucleatum*.

We further validated changes in inflammatory factors IL-1β, IL-6, and IL-8 in the GECs with ELISA. The results showed that in the *F. nucleatum* group, IL-1β, IL-6, and IL-8 were much higher in the supernatant than in the control and AKK groups; IL-6 was twice as high as that in the Fn+AKK group, and IL-1β and IL-8 were approximately 1.5 times as high as that in the Fn+AKK group (Fig. 3j–l), indicating that *F. nucleatum* promoted the secretion of inflammatory factors in GECs, while *A. muciniphila* inhibited this response, consistent with the qPCR results.

### *A. muciniphila* inhibited *F. nucleatum*-induced periodontitis in BALB/c mice

These findings suggested that *A. muciniphila* could inhibit *F. nucleatum*-induced inflammation of GECs. Next, we explored whether *A. muciniphila* can perform the same role in vivo. BALB/c mice were respectively and together inoculated with *A. muciniphila* and *F. nucleatum* to observe the bacterial community structure, alveolar bone loss, bone volume fraction, and soft and hard tissue inflammation to determine the role of *A. muciniphila*. After bacterial inoculation four days a week for four consecutive weeks, qPCR for oral plaque samples of mice showed that *F. nucleatum* and *A. muciniphila* successfully colonized the oral cavity (Supplementary Fig. 3a, b).

**Fig. 4:**
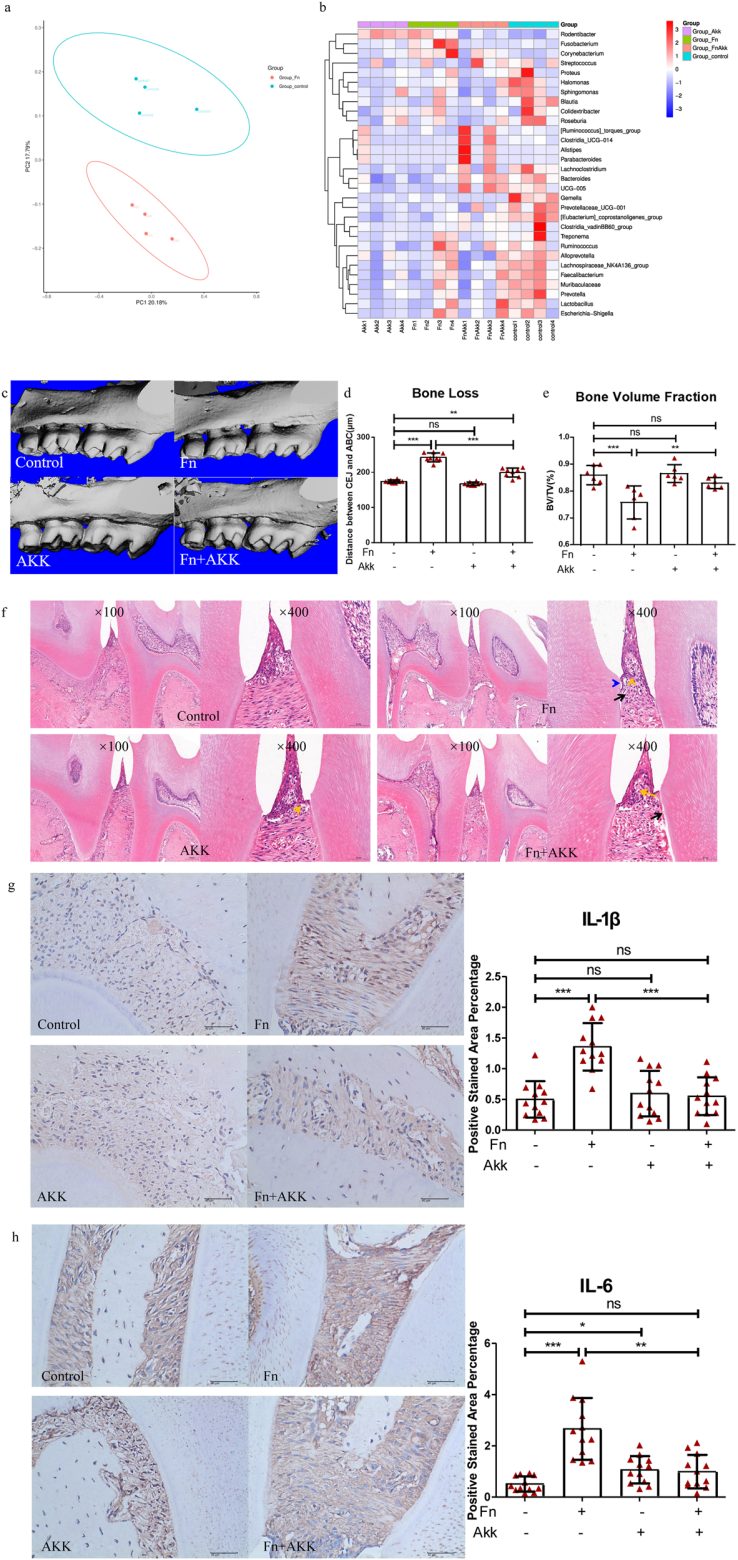
*A. muciniphila* inhibited *F. nucleatum*-induced periodontitis in BALB/c mice. **a** PCoA analysis of oral plaque samples. Fn vs. control (*n* = 4). **b** The heat map of oral plaque sample community structure in mice: all four groups **c** 3D reconstruction of the maxilla. **d** Alveolar bone loss in the mesial aspect of the maxillary first molar, *n* = 10. **e** Bone volume fraction at the bifurcation of the root of the maxillary first molar. **f** H&E staining images of the mandible (×100 and ×400), scale = 100 μm and 50 μm. Yellow arrows: inflammatory cell infiltration; black arrows: disordered fibrous arrangement; blue arrows: stripped junctional epithelium. **g**, **h** Immunohistochemical staining images (×400) of mandibular and the percentage of protein-positive area: IL-1β and IL-6, scale = 40 μm. Data are shown as the mean ± SD. Statistical significance was determined by Wilcoxcon rank-sum test, One-way analysis of variance (ANOVA) and Student–Newman–Keuls test. *P < 0.05, **P < 0.01, ***P < 0.001, ns not significant.

Mice oral plaque was used for 16s rRNA sequencing. Although there were no significant differences, the observed species and PD whole tree indices showed the least species richness in the Fn group (Supplementary Fig. 3c, d). In contrast, species richness and uniformity of the AKK and Fn+AKK groups were slightly higher than the control and Fn groups (Supplementary Fig. 3d), indicating that *F. nucleatum* might have reduced oral species diversity, while *A. muciniphila* maintained and improved oral species diversity. As indicated by PCoA analysis, the oral community structure of the control group was significantly different from the Fn group (Fig. 4a) but was similar to the AKK group (Supplementary Fig. 3e). There were no significant differences between the Fn+AKK, Fn, and control groups (Supplementary Fig. 3f). The heat map of the community structure showed that the abundance of *F. nucleatum* was the highest in the Fn group (Fig. 4b), indicating that *A. muciniphila* might have inhibited the colonization of *F. nucleatum* in the oral cavity in the Fn+AKK group. In addition, *A. muciniphila* also inhibited the growth of other periodontal pathogens such as *Prevotella*, *Treponema*, and *Campylobacter* (Fig. 4b).

After being sacrificed, we used the maxillary bones of mice for micro-CT analyses. Concerning alveolar bone loss, the Fn group exhibited the highest bone loss, and the AKK group was similar to the control group. The Fn+AKK group exhibited less bone loss than the Fn group but more than that in the control group (Fig. 4c, d). The bone volume fraction at the root bifurcation of the maxillary first molar was the least in the Fn group (Fig. 4e), indicating that *F. nucleatum* might have promoted alveolar bone resorption while *A. muciniphila* might have inhibited it.

HE staining was performed to observe the periodontal inflammatory response. There was no apparent inflammation in the control group, and a few inflammatory cells were observed in the gingiva of the AKK group. In contrast, there were numerous inflammatory cells, a disordered arrangement of periodontal membrane fibers, and stripped junctional epithelium in the Fn group. However, the Fn+AKK group showed milder inflammation than the Fn group (Fig. 4f), indicating that *F. nucleatum* might have exerted a severe inflammatory effect on the periodontal tissue, while *A. muciniphila* might have inhibited it. The immunohistochemical staining results showed that the proportion of IL-1β-positive area (1.36% vs. about 0.5%) and IL-6-positive area (2.66% vs. 0.51%, 1.06%, and 0.99%) in the Fn group were the highest (Fig. 4g, h), indicating that *F. nucleatum* infection might have led to elevated levels of IL-1β and IL-6 proteins in mouse periodontal tissues, and *A. muciniphila* might have inhibited it, consistent with HE staining results.

## Discussion

*F. nucleatum* is the dominant pathogen in periodontitis. It promotes the formation and maturation of plaque biofilm through copolymerization to develop periodontitis in conjunction with the red complex^7–9^. It also mediates the relationship between periodontitis and systemic diseases such as colorectal cancer^10–12^. Considering the critical periodontal and systemic pathogenic effects of *F. nucleatum*, inhibiting its growth and virulence gene expression is significant for periodontal and general health.

In recent years, considering the limitations of periodontal scaling and root planing^39^ and microbial resistance and oral microbiome imbalance caused by antibiotics, probiotics such as *Lactobacillus*, *Bifidobacterium*, and *A. muciniphila* have been introduced into the treatment of periodontitis^13,19,40–42^. These probiotics can produce some antibacterial products, regulate the host immune response, and directly interact with pathogens to inhibit the progression of periodontitis. *A. muciniphila* has attracted attention as an intestinal probiotic in recent years^30,31^. Its abundance is inversely correlated with various diseases. It can also be detected in the saliva^35^, and researchers have verified the safety of *A. muciniphila* for oral administration^24^. Therefore, we explored its role in periodontitis.

**Fig. 5:**
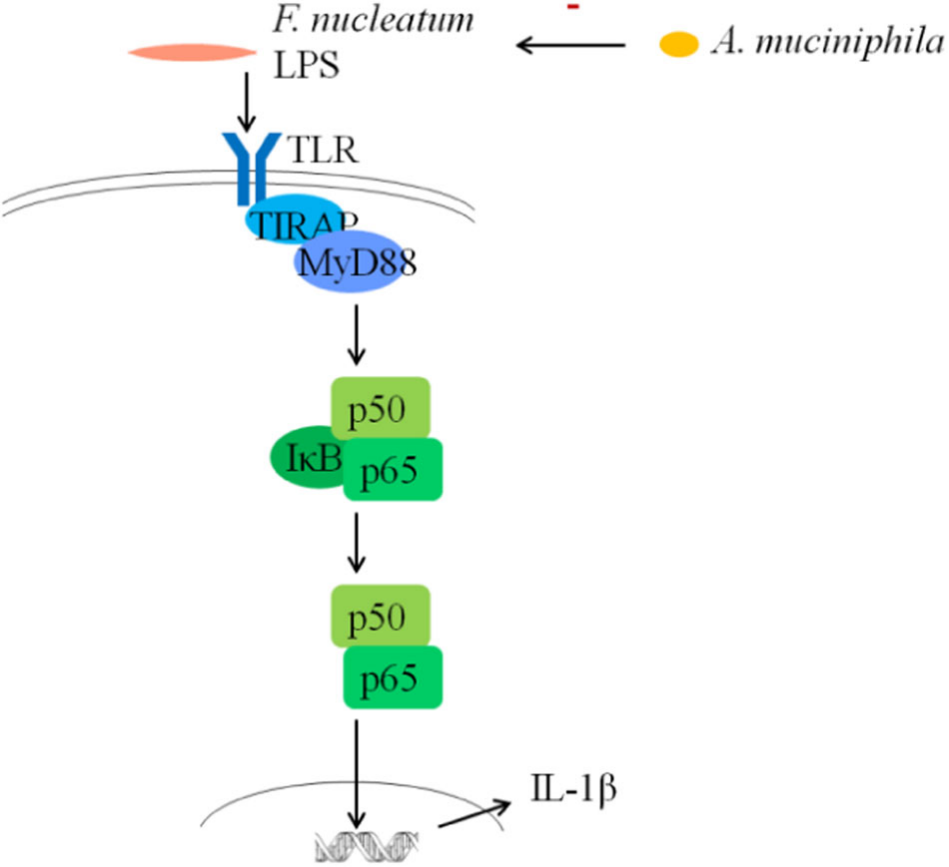
Possible mechanisms of *F. nucleatum* on human GECs and inhibition of the inflammatory effect by *A. muciniphila*.

Previous studies have shown that *F. nucleatum* can invade GECs with the help of adhesins such as FadA^43–45^ and generate LPS to activate the TLR/MyD88/NF-κB pathway^46,47^ which in turn produces IL-1β^48,49^. Our study found that *A. muciniphila* inhibited the expression of virulence factor FadA of *F. nucleatum* at 24 h, 48 h, and 72 h (Fig. 2a–c). Furtherly, the transcriptome and qPCR analysis of the GECs indicated that inflammatory factor-related genes, including TLRs, MyD88, NF-κB1, IL-1β and so on, were significantly downregulated in the Fn+AKK group compared with the Fn group (Fig. 3a). qPCR of representative factors of the NF-κB pathway showed, in the Fn+AKK group, TLR-2, TLR-4, MyD88, and NF-κB1 expressed approximately half as much as those in the Fn group (Fig. 3b–e). In addition, inflammatory factors of periodontitis, such as IL-1β, IL-6, IL-8, and TNF-α also significantly reduced in the Fn+AKK group (Fig. 3f–i). In the mice model, *A. muciniphila* also reduced the IL-1β and IL-6 levels inuduced by *F. nucleatum* (Fig. 4g, h). According to these results, we thought that *A. muciniphila* could inhibit the growth and the expression of virulence genes of *F. nucleatum*, especially the FadA gene, then downregulated the TLRs/MyD88 signaling pathway to furtherly inhibit the activation of NF-κB pathway, and finally reduced the productions of inflammatory factors, such as IL-6, IL-8 and IL-1β (Fig. 5). Finally, we showed that *A. muciniphila* could inhibit the colonization of *F. nucleatum* in the oral cavity and may reverse the microbial structure disorders through the *F. nucleatum*-induced mouse periodontitis model. In addition, *A. muciniphila* can also inhibit alveolar bone loss and inflammation of soft and hard tissues.

Interestingly, we found that in addition to the growth and virulence of *F. nucleatum*, *A. muciniphila* also inhibited the growth of other periodontal pathogens such as *Peurella*, *Treponemalia*, and *Campylobacter* (Fig. 4b). We suspect that this may be related to the bridging role of *F. nucleatum* in the formation of dental plaque. *F. nucleatum* has a narrow rod-like structure and can express a variety of adhesins. On the one hand, it combines initially colonized pathogenic bacteria such as *Streptococcus mutants* (Sm) on the tooth surface^50–52^, relying on arginine-inhibitable adhesin (RadD), adherence-inducing determinant 1 (Aid1), and coaggregation-mediating-protein-A (CmpA). On the other hand, *F. nucleatum* can be copolymerized with later colonized pathogenic bacteria such as *Porphyromonas gingivalis* (Pg)^40,42^ through adhesin RadD, porin FomA, and fatty acid-binding protein 2 (Fap2). *F. nucleatum* can copolymerize many cariogenic and periodontal pathogenic bacteria, promoting the formation and maturation of dental plaque, the initiating factor of these two diseases. In the development of periodontitis, *F. nucleatum* can copolymerize bacteria such as Pg^41^, promoting their periodontal pathogenic effects. *A. muciniphila* may inhibit the formation of plaque biofilms by inhibiting the copolymerization of *F. nucleatum* and inhibiting the pathogenicity of *F. nucleatum* and other periodontal pathogenic bacteria. Nevertheless, we suspect that *A. muciniphila* may have direct or indirect inhibitory effects on other periodontal pathogens, necessitating further experiments.

In addition to periodontal pathogenicity, *F. nucleatum* mediates the relationship between periodontitis and systemic diseases such as colorectal cancer^23,26,28^. *F. nucleatum* can bind to E-cadherin through FadA, activate β-catenin signaling, and differentially regulate inflammatory and carcinogenic responses to stimulate the growth of colorectal cancer cells^53^. It can also activate the nuclear factor NF-κB by activating the TLR4/MyD88 signaling pathway, upregulating the expression of miRNA-21, regulating the expression of RASA1, and activating the MAPK pathway, finally promoting colorectal cancer cell proliferation and tumor development^54^. In addition, *Fusobacterium nucleatum* can induce the secretion of tumor-associated cytokines and inflammatory factors, such as IL-21/22/31 and CD40L, activating JAK/STAT and MAPK/ERK signaling pathways to promote tumorigenesis^55^. Periodontitis increases the risk of colorectal cancer; the present study found that the inhibitory effect of *A. muciniphila* on *F. nucleatum*, especially its vital virulence factor FadA, is of great significance for preventing and treating periodontitis and colorectal cancer.

Previous studies have shown that *A. muciniphila* can improve host metabolic disorders, regulate immune function, and inhibit intestinal inflammation through P9 protein, outer membrane protein Amuc-1100, or the production of specific metabolites such as short-chain fatty acids. Although our study showed that *A. muciniphila* could inhibit the progression of periodontitis by inhibiting *F. nucleatum*, the particular components of its inhibitory effect are still unclear, necessitating further research.

In summary, our study showed that *A. muciniphila* could inhibit the growth and virulence gene expression of *F. nucleatum*. It inhibited the secretion of inflammatory factors and expression of the TLR/MyD88/NF-κB pathway caused by *F. nucleatum* to inhibit its inflammatory effect on GECs, which was validated in a mouse model of periodontitis. Our study provides new insights into the prevention and treatment of periodontitis.

## Methods

### Bacterial culture

The Chinese Oral Microbiology Resource Database and West China Second Hospital of Sichuan University provided *F. nucleatum* (ATCC 25586) and *A. muciniphila* (DSM 22959).

*F. nucleatum* and *A. muciniphila*^56^ were cultured in a 37 °C multifunctional anaerobic incubator (GENE SCIENCE, USA) (80% N_2_, 10% H_2_, 10% CO_2_) in BHI medium (BD, USA) containing Hemin (5 μg/mL, J&K Scientific, China), vitamin K1 (1 μg/mL, J&K Scientific, China) and 3% MUCIN (Type III) (Sigma, USA) for 48 h, respectively. In the BHI-Hemin-VK medium, the multifunctional enzyme-linked immunoassay (Molecular Devices, USA) showed *F. nucleatum* at approximately 1 × 10^9^ CFU/mL at a wavelength of 600 nm and OD = 0.5. In the BHI-Mucin medium, *A. muciniphila* concentration was about 1 × 10^9^ CFU/mL at a wavelength of 600 nm and OD = 0.4

1 × 10^8^ CFU/mL of *F. nucleatum* and 1 × 10^7^ CFU/mL of *A. muciniphila* were co-cultured in BHI-Hemin-VK medium at 1:1 volume in an anaerobic environment (80% N_2_, 10% H_2_, 10% CO_2_) at 37 °C. The bacteria in each group were collected for 1‒3 days and transferred to wall-breaking tubes with broken-wall beads. Then, 1 mL of Trizol was added to the liquid nitrogen cryogenic disruption cell apparatus (Bertin, USA) to break nitrogen-quick-frozen bacteria, followed by storage at −80 °C for subsequent RNA extraction.

Glass slides were coated with sterile saliva for 24 h and placed in a six-well plate with 2 mL of BHI-Hemin-VK medium. The starting concentrations of *F. nucleatum* and *A. muciniphila* were 1 × 10^9^ and 1 × 10^8^ CFU/mL, respectively. Then, 100 μL of the bacterial solution was added and cultured for 1‒3 days in the Fn and Fn+AKK groups (a volume of 1:1).

### Standard curve

The second generation 1 × 10^9^-CFU/mL *F. nucleatum* and *A. muciniphila* was diluted to the 1/10, 1/100, 1/1000, and 1/10^4^ initial concentration, 1 mL of which was transferred to a 1.5-mL EP tube, using the bacterial genome DNA extraction kit (Tiangen, China) according to the instructions to extract bacterial DNA. Then their 16S rRNA gene-specific primer sequence **(**Table 1**)** was prepared in a 20-μL reaction system according to the TaKaRa TB Green® Premix Ex TaqTM II specification using a real-time fluorescence quantitative PCR instrument (Roche, Switzerland) to run the qPCR program and obtain a standard curve whose ct value corresponded to the concentration.

**Table 1:** Bacterial 16S rRNA gene-specific primers.

Primer	Sequence
Fn-Forward	5′-CAACCATTACTTTAACTCTACCATGTTCA-3′^58^
Fn-Reverse	5′-GTTGACTTTACAGAAGGAGATTATGTAAAAATC-3′^58^
Akk-Forward	5′-CAGCACGTGAAGGTGGGGAC-3′^59^
Akk-Reverse	5′-CCTTGCGGTTGGCTTCAGAT-3′^59^

### Cell culture

Human gingival epithelial cells (C1052) were provided by Shanghai Chunmai Biotechnology Co., Ltd., China. It was cultured in a DMEM medium (Gibco, USA) containing 10% FBS (Gibco, USA) and 1% penicillin/streptomycin (Gibco, USA) (5% CO_2_) at 37 °C. Human GECs were tiled in a six-well plate with 3–5 × 10^5^/well, and after adhering, they were divided into the control, Fn, AKK, and Fn+AKK groups. Except for the control group, the remaining three were resuspended with 10% FBS DMEM medium, and the cells were infected (MOI = 250) and cultured for 24 h (5% CO_2_, 37 °C). After centrifuging at 1000*g* for 20 min, the supernatant was collected and stored at −80 °C for ELISA. The cells were washed with PBS, and 1 mL of Trizol was added, quickly frozen with liquid nitrogen, and saved at −80 °C for RNA extraction.

### Animals

Animal experiments were conducted after approval by the Ethics Committee of West China Stomatology Hospital of Sichuan University. Five-week-old male SPF-grade BALB/c mice were purchased from Chengdu Dashuo Animal Center and acclimatized to the environment for one week. Water was treated with trimethoprim (0.17 mg/mL) and sulfamethoxazole (0.87 mg/mL) for one week^40^. Five days after antibiotic treatment^50^, the mice were randomly divided into four groups (control, Fn, AKK, and Fn+AKK groups, *n* = 10). Subsequently, 2% CMC solution was configured with PBS as a bacteria-resuspended solvent. The bacteria were applied to the oral cavities of the mice (10^8^ CFU/mL, 0.2 mL/day, four days/week). The color, morphology, and texture changes of mice’s gingiva were examined weekly to determine the inflammatory state. After eight weeks, cotton swabs were used to collect oral plaque samples and collect maxillas and mandibles to fix in 4% paraformaldehyde solution. The maxillas were used for micro-CT analyses, and the mandibles were used for H&E staining analyses to observe inflammation in soft and hard tissues.

### Electron microscopic scanning of biofilms

The co-cultured glass slides were washed 2‒3 times with PBS. The planktonic microorganisms were washed away and fixed overnight using 2.5% glutaraldehyde at 4 °C. Then, 30%, 50%, 70%, 80%, 90%, 95%, and absolute ethanol gradients were used sequentially. Each concentration was treated for 10 min and dried using a gold jet after scanning electron microscopy (Leica, China) for observation.

### RNA isolation and quantitative polymerase chain reaction

The total RNA of co-cultured bacteria was extracted by the Trizol method, and NANODROP ONEC was used to determine RNA concentration and quality. Reverse transcription of RNA using PrimeScriptTM RT reagent Kit with gDNA Eraser (TaKaRa, Japan) according to the manufacturer’s instructions yielded cDNA, which was stored at −20 °C. *F. nucleatum* virulence gene primers (Table 2) were designed. The qPCR method was the same as before, and 2^-ΔΔCt^ calculated the relative expression.

**Table 2:** *F. nucleatum* virulence gene-specific primers.

Primer	Sequence
FadA-Forward	5′-TGCAGCAAGTTTAGTAGGTG-3′^60^
FadA-Reverse	5′-CATTGTAAACTTGTTCATTTTGT-3′^60^
Fap2-Forward	5′-AAAATTGGAGCAACAGGAGGA-3′^60^
Fap2-Reverse	5′-TTCAGAGGCAATAGCGACAAC-3′^60^
FomA-Forward	5′-AGAGTTTGATCCTGGCTCAG-3′^61^
FomA-Reverse	5′-GTCATCGTGCACACAGAATTGCTG-3′^61^
Aid1-Forward	5′-TACAGGAGGTGCCGTAGCAG-3′^62^
Aid1-Reverse	5′-TTTTTGTTAATTCTCCAGCTCCA-3′^62^
CmpA-Forward	5′-TTGGGATCAAGGAAAACATCAATTAGG-3′^61^
CmpA-Reverse	5′-ATAATTCCTTTATTATCTCCCATATAAGCAATACC-3′^61^
RadD-Forward	5′-GGATTTATCTTTGCTAATTGGGGAAATTATAG-3′^63^
RadD-Reverse	5′-ACTATTCCATATTCTCCATAATATTTCCCATTAGA-3′^63^

Total RNA in cells was extracted by the Trizol method, Nanodrop2000 was used for concentration and purity detection, and agarose gel electrophoresis was used to detect RNA integrity. Illumina Novaseq 6000 sequencing platform was used for double-ended sequencing, with SeqPrep for raw sequencing data quality control, HiSat2 for reference genome comparison, RSEM for gene expression, DESeq2 for differential expression calculation, Goatools for GO enrichment analysis, and KOBAS for KEGG enrichment analysis (primer design is shown in Table 3).

**Table 3:** Inflammation-related gene-specific primers for human gingival epithelial cells.

Primer	Sequence
TLR-2-Forward	5′-GATGCCTACTGGGTGGAGAA-3′^64^
TLR-2-Reverse	5′-CGCAGCTCTCAGATTTACCC-3′^64^
TLR-4-Forward	5′-AACCATCCTGGTCATTCTCG-3′^64^
TLR-4-Reverse	5′-CGGAAATTTTCTTCCCGTTT-3′^64^
MyD88-Forward	5′-TAAGAAGGACCAGCAGAGCC-3′^65^
MyD88-Reverse	5′-CATGTAGTCCAGCAACAGCC-3′^65^
NF-κB1-Forward	5′-CCTGGATGACTCTTGGGAAA-3′^64^
NF-κB1-Reverse	5′-CTTCGGTGTAGCCCATTTGT-3′^64^
IL-1β-Forward	5′-CACGCTCCGGGACTCACAGC-3′^66^
IL-1β-Reverse	5′-CTGGCCGCCTTTGGTCCCTC-3′^66^
IL-6-Forward	5′-CGCCCCACACAGACAGCCAC-3′^66^
IL-6-Reverse	5′-AGCTTCGTCAGCAGGCTGGC-3′^66^
IL-8-Forward	5′-TTTCTGATGGAGAGAGCTCTGTCTGG-3′^66^
IL-8-Reverse	5′-AGTGGAACAAGACTTGTGGATCCTGG-3′^66^
TNF-α-Forward	5′-TTCTGCCTGCTGCACTTTGGA-3′^66^
TNF-α-Reverse	5′-TTGATGGCAGAGAGGAGGTTG-3′^66^
GAPDH-Forward	5′-ACCACAGTCCATGCCATCACTGC-3′^66^
GAPDH-Reverse	5′-TCCACCACCCTGTTGCTGTAGC-3′^66^

### Analyses of supernatant proinflammatory cytokine

After co-culturing GECs with bacteria, the supernatant was analyzed using ELISA Kit for interleukin-1β/6/8 (Cloud-Clone Corp, USA) for ELISA experiments according to the manufacturer’s instructions.

### Micro-computed tomography analyses of alveolar bone

Micro-CT scans of the maxillas of mice in each group were performed (SCANCO Medical AG, Switzerland) with a voxel resolution of 10 μm. Sagittal images of the middle tooth were selected, and the distance between the alveolar crest and enamel junction was measured to quantify the loss of the distal aspect of the first molar and the mesial aspect of the second molar. Bone volume fraction (BV/TV) analysis per tissue volume in the region of interest was performed using CT-Analyser 1.13 software (Bruker). The area of interest was the rectangular area of approximately 5.2 mm^2^ of the alveolar bone at the mesial and distal root bifurcation of the maxillar first molar^57^.

### Histologic analyses of alveolar bone

Chengdu Lilai Biotechnology Co., Ltd carried out histopathological testing. HE staining revealed inflammation of the gingiva and alveolar bone, and immunohistochemical staining detected IL-1β and IL-6 protein content in the periodontal tissues of mice.

### DNA sequencing and analyses

Shanghai OE Biomedical Technology Co., Ltd. used Illumina MiSeq or NovaSeq sequencing to generate the original double-ended sequence and sequenced the 16S rRNA amplicon (V3-V4 region). The clean tags data were distributed between 72684 and 76924. Chimerism was removed to obtain valid tags distributed between 60739 and 72044. The average length of the valid tags was distributed between 414.07 and 424.22 bp; the number of OTU of each sample was distributed between 724 and 1406. QIIME and MG-RAST were used to perform classification notes based on the Silva database, taxonomic comparisons at the genus level, β-diversity analysis (principal coordinate analysis), and variance analysis (i.e., ANOSIM and Adonis).

### Statistical analysis

One-way analysis of variance (ANOVA) was performed using GraphPad Prism 7 software to compare group-to-group differences, and the two-by-two comparisons were performed using the Student–Newman–Keuls test method at a statistical significance of *P* < 0.05.

## Supplementary information

revised supplementary materials

nr-reporting-summary

## Data Availability

All data generated or analyzed during this study are included in this published article (and its supplementary information included in this published article and its supplementary information files). The sequencing data from this study have been submitted to NCBI’s Sequence Read Archive under accession no. PRJNA918716 and no. PRJNA917240.
