# Supplementary material for: Akkermansia muciniphila inhibited the periodontitis caused by Fusobacterium nucleatum
Source: NPJ Biofilms Microbiomes. 2023 Jul 17;9:49. doi: 10.1038/s41522-023-00417-0 (PMC10352368; doi:10.1038/s41522-023-00417-0)
Supplement: Supplementary file 1 — revised supplementary materials [file 41522_2023_417_MOESM1_ESM.pdf]

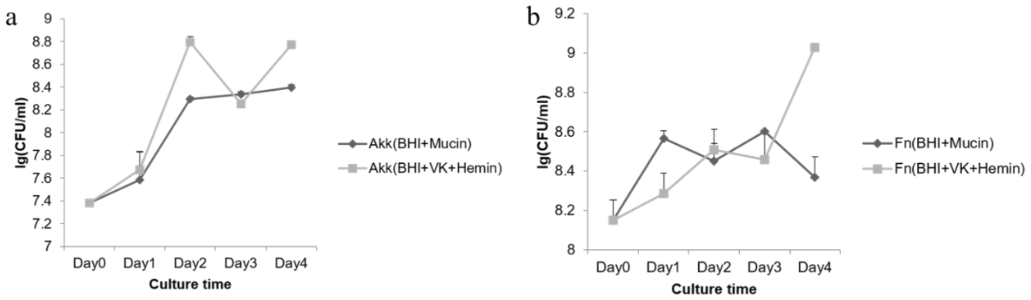

Supplementary figure 1 A. muciniphila inhibits the growth of F. nucleatum in a planktonic or biofilm state. a. The amount of A. muciniphila in two media. **b.** The amount of F. nucleatum in two media. Data are shown as the mean ± SD.

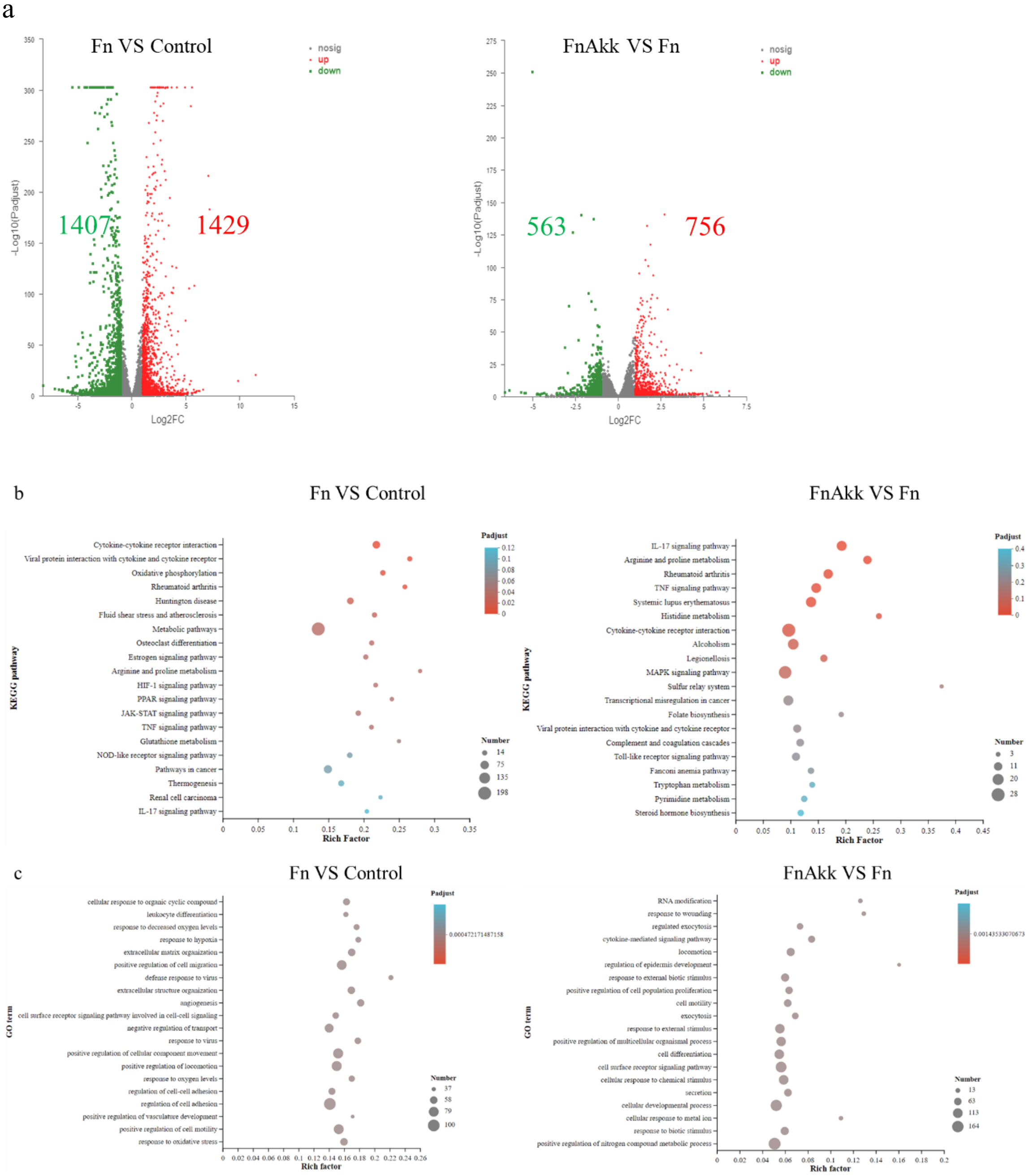

Supplementary figure 2 A. muciniphila inhibits the inflammatory effect on

gingival epithelial cells caused by F. nucleatum via inhibiting expression of TLR/MyD88/NF-κB pathways and inflammatory factors secretion. a. Gene expression of human GECs treated by bacteria for 24h (abscissa and ordinate values are logarithmic: multiple of the difference in gene expression, ordinate: statistical test value of the difference in gene expression, red dot: significantly upregulated gene, green dot: significant downregulation gene, gray dot: non-significantly different gene)； **b-c.** KEGG and GO functional enrichment analysis of human GECs treated by bacteria for 24h (abscissa: Rich Factor, ordinate: KEGG and GO term, color represents P_{adjust}, bubble size represents the number of genes in the pathway);

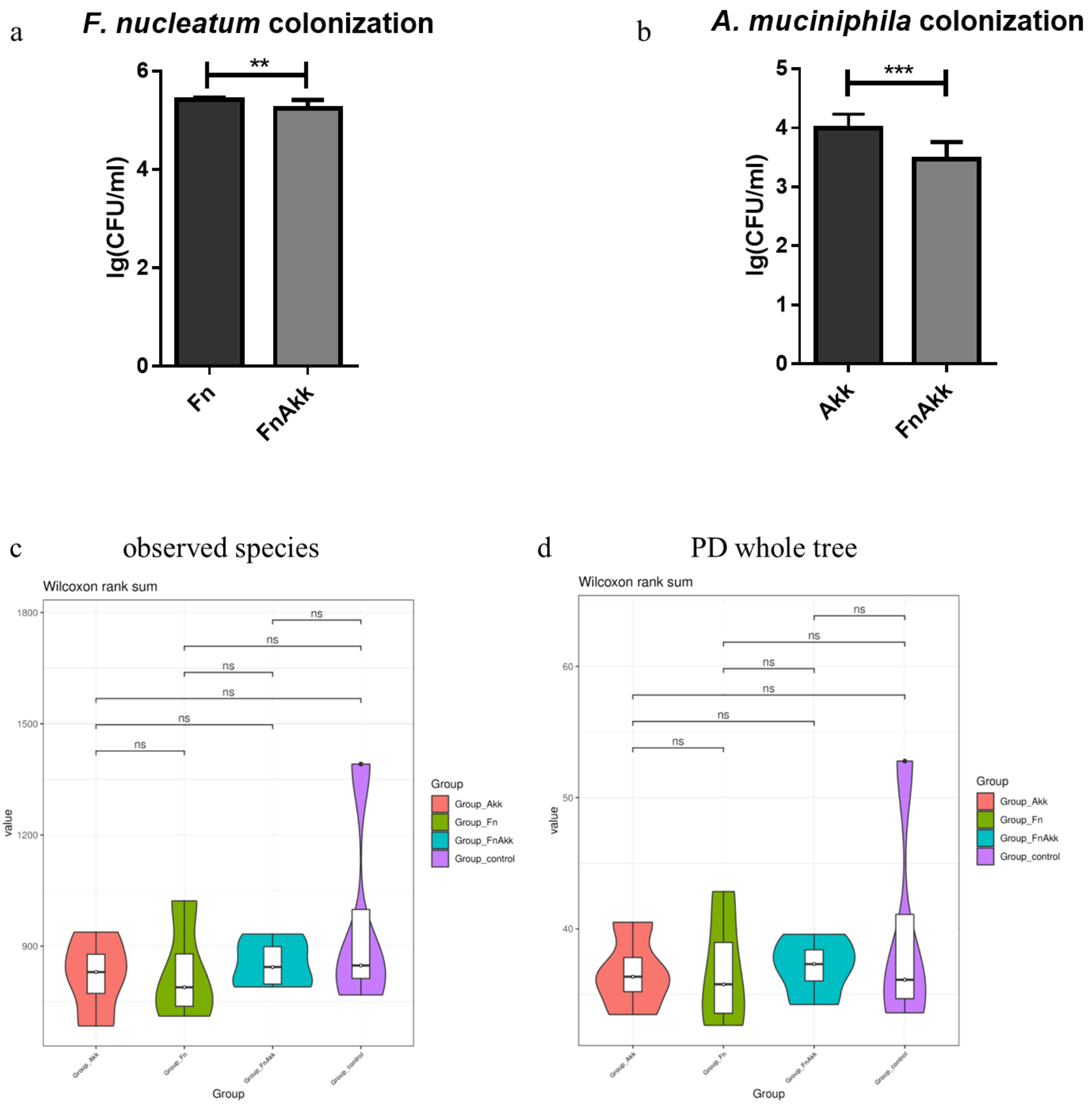

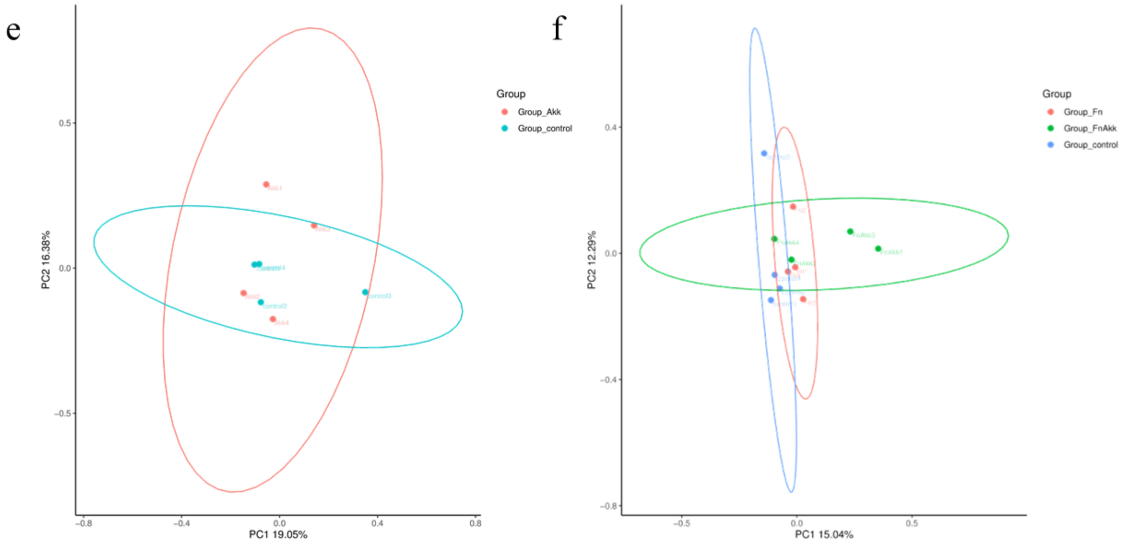

Supplementary figure 3. A. muciniphila inhibits F. nucleatum-induced periodontitis in BALB/c mice. a-b. Colonization of F. nucleatum**(a)** and A. muciniphila**(b)** in the Fn group and the Fn+AKK group; **c-d.** α diversity analysis of oral plaque samples: observed species**(c)** and PD whole tree**(d)**; **e-f.** PCoA analysis of oral plaque samples. AKK vs Control **(e)**; Fn +AKK, Fn vs Control **(f).** Data are shown as the mean ± SD. Statistical significance was determined by Wilcoxon rank-sum test and Student-Newman-Keuls test. *: $P<0.05$, **: $P<0.01$, ***: $P<0.001$, ns: not significant.
